# Mating Systems, Reproductive Success, and Sexual Selection in Secretive Species: A Case Study of the Western Diamond-Backed Rattlesnake, *Crotalus atrox*

**DOI:** 10.1371/journal.pone.0090616

**Published:** 2014-03-05

**Authors:** Rulon W. Clark, Gordon W. Schuett, Roger A. Repp, Melissa Amarello, Charles F. Smith, Hans-Werner Herrmann

**Affiliations:** 1 Department of Biology, San Diego State University, San Diego, California, United States of America; 2 Department of Biology and Center for Behavioral Neuroscience, Georgia State University, Atlanta, Georgia, United States of America; 3 The Copperhead Institute, Spartanburg, South Carolina, United States of America; 4 Chiricahua Desert Museum, Rodeo, New Mexico, United States of America; 5 National Optical Astronomy Observatory, Tucson, Arizona, United States of America; 6 Social Snakes, Tucson, Arizona, United States of America; 7 Department of Biology, Wofford College, Spartanburg, South Carolina, United States of America; 8 School of Natural Resources and the Environment, University of Arizona, Tucson, Arizona, United States of America; Université de Sherbrooke, Canada

## Abstract

Long-term studies of individual animals in nature contribute disproportionately to our understanding of the principles of ecology and evolution. Such field studies can benefit greatly from integrating the methods of molecular genetics with traditional approaches. Even though molecular genetic tools are particularly valuable for species that are difficult to observe directly, they have not been widely adopted. Here, we used molecular genetic techniques in a 10-year radio-telemetric investigation of the western diamond-backed rattlesnake (*Crotalus atrox*) for an analysis of its mating system and to measure sexual selection. Specifically, we used microsatellite markers to genotype 299 individuals, including neonates from litters of focal females to ascertain parentage using full-pedigree likelihood methods. We detected high levels of multiple paternity within litters, yet found little concordance between paternity and observations of courtship and mating behavior. Larger males did not father significantly more offspring, but we found evidence for size-specific male-mating strategies, with larger males guarding females for longer periods in the mating seasons. Moreover, the spatial proximity of males to mothers was significantly associated with reproductive success. Overall, our field observations alone would have been insufficient to quantitatively measure the mating system of this population of *C. atrox*, and we thus urge more widespread adoption of molecular tools by field researchers studying the mating systems and sexual selection of snakes and other secretive taxa.

## Introduction

The long-term study of individual organisms in nature plays a central role in our understanding of ecology and evolution [1]. Despite logistical difficulties, long-term investigations are necessary to interpret the processes affecting survival and reproduction played out over the course of multiple years, or even decades [2]. However, such studies are few and there is a strong taxonomic bias. For example, all of the studies cited in Clutton-Brock and Sheldon [1] concern mammals and birds. This bias toward endotherms is most likely related to the fact that these species can be observed in nature more easily. Consequently, a large number of terrestrial vertebrates remain severely understudied [3], [4]. These species often exhibit some combination of being small, cryptic, secretive, nocturnal, and sedentary. Hence, in our view, long-term studies on these taxa may disproportionately benefit from techniques that allow researchers to infer patterns of intraspecific interactions that are difficult or not possible to document by way of direct observation.

Molecular genetics can be used to genotype individuals, measure relatedness, determine parentage, and unravel patterns of social structure and mating systems that would otherwise be extremely difficult to document [5], [6]. Despite the obvious benefits of this approach in addressing questions in behavioral ecology and evolution, molecular genetic tools have not been widely adopted for some taxa. For example, the mating systems of hundreds of avian species have been quantified and characterized using molecular genetic approaches [7], [8]. In sharp contrast, although Gibbs and Weatherhead [9] highlighted the utility of molecular genetics to revolutionize our understanding of snake mating systems over a decade ago, only a handful of subsequent studies have used genetic tools in combination with field studies to provide detailed characterizations of sexual behavior and mating systems in wild snakes (see [10]-[14]).

Large species of snakes, such as many viperids, boids and pythonids, are ecologically and economically important, but most are unstudied in nature. They are important predators in many ecosystems and can occur in much higher densities than their endothermic counterparts [15]. Yet, despite sedentary lifestyles, their generally secretive habits make most species difficult to observe directly in nature [9], [16], [17]. However, they are ideal for long-term, individual-based studies employing radio-telemetry, and their relatively high population densities permit the study of large numbers of individuals in relatively small areas [18], [19]. Most ecological studies on viperids, boids and pythonids have focused on population-level measures of movements, habitat use, survival, and demography. Studies of individual behaviors and intraspecific interactions are less common than in other vertebrate groups, probably because acquiring such data through direct observation can be prohibitively time consuming. Use of molecular genetic tools in field studies of snakes and other ectotherms can be both an alternative and adjunct to direct observation [6], [9], [20], [21].

Here, we demonstrate the utility of combining traditional field techniques with molecular genetics in a long-term study of a North American pitviper (Serpentes; Viperidae). Specifically, we quantified the behavioral and genetic mating system of western diamond-backed rattlesnakes (*Crotalus atrox*) in southern Arizona for 10 consecutive years (2001-2010) by incorporating direct observation of radio-tagged individuals, opportunistic observation, and tissue sampling (blood, shed skins of adults and neonates) of both radio-tagged and incidental subjects. We used a polymorphic panel of 27 microsatellite markers to genotype all individuals sampled over the course of our decade long study, and infer patterns of parentage and mating from the genetic analyses. We asked the following questions: 1) Are litters sired by multiple males, and if so, what is the frequency of multiple paternity? 2) Do larger males sire more offspring? 3) Are genetic fathers the same males we see attending and courting females in the mating seasons prior to parturition? 4) Is litter size affected by level of paternity (i.e., single father *vs*. multiple fathers)? 5) Do males sire more offspring when they are spatially closer to mothers?

## Materials and Methods

### Ethics Statement

The Institutional Animal Care and Use Committee (IACUC) of Arizona State University approved this study (protocol 98-429R), and appropriate scientific permits were obtained from the Arizona Game and Fish Department.

### Study system

We studied a single population of *C. atrox* in southern Arizona (USA) for 10 consecutive years (2001–2010). The research site, the Suizo Mountains (SMs) located in Pinal County (Arizona), is 40 km SSE of the city of Florence, 8 km W of State Route 79 [22], [23]. We measured, weighed, and collected blood samples for DNA analysis from all adults encountered at this site. We also surgically implanted radio-transmitters into a subset of adults. We tracked these individuals by foot minimally 2-4 times per month from 2001 to 2010. Tracking was increased substantially (sometimes daily or twice daily) from early August through mid-September, the period of birthing in *C. atrox* [22], [23]. For each radio-tracked subject located, UTM coordinates were recorded using a hand-held Global Positioning System (GPS) unit.

### Research site

The focal area encompasses 3 km^2^ at the western edge of the SMs (32°40′08′ ′N, 111°07′22′ ′W, Conus 27). The SMs have a summit elevation of 947 m. The region is designated as Sonoran Desert, Arizona Upland Desert-Scrub subdivision [24], [25]. In addition to mountainous terrain, the general topography is bajada and desert flats, intersected by dry washes of varying sizes. Annual rain patterns of the Sonoran Desert are bimodal [26], [27]. Gentle to moderate broad frontal storms occur from late fall to early spring (November–March), and strong to violent, often localized convective storms occur from mid- to late summer (early July to mid-September), the period of the North American monsoon [26]. Free water is rarely available and highly unpredictable at the SMs.

### Ecology

The western diamond-backed rattlesnake (*C. atrox*) is a large-bodied pitviper (Serpentes: Viperidae). Throughout its wide geographic distribution in the western United States and Mexico [28], [29], *C. atrox* exhibits minor morphological variation [30] and shallow genetic (mtDNA) differences [31], yet adult body size varies significantly [30]. In Arizona, adult body sizes of adult *C. atrox* in different populations show significant differences and male-biased sexual size dimorphism [32]. Arizona has broad physiographic structure and multiple biotic communities within a relatively narrow latitudinal range [24], and *C. atrox* occupies the southern half of the state and most of its biotic regions [29]. Adults of both sexes in *C. atrox* exhibit significantly larger snout-vent length (SVL) in regions of Arizona that are wetter and cooler [32], two variables that are associated with increases in their common prey, such as small mammals, birds and lizards [33]. Presumably, this is linked to increases in prey opportunities to acquire sufficient body reserves, especially in females, for reproduction and growth [22], [23], [34]. In some regions, *C. atrox* is extremely abundant and frequently reported as the dominant snake species, sometimes even the dominant vertebrate predator [15].

### Phenology of mating behaviour

Knowledge of the behavioral and genetic mating system of *C. atrox* is in its infancy, and no study to date has robustly characterized male and female mating strategies and quantified reproductive success in nature. However, qualitative components of the mating system of *C. atrox* are characteristic of other species of North American rattlesnakes and other pitvipers [35]. In *C. atrox* and other pitviper species, there are two distinct mating seasons that occur prior to the period of ovulation (for recent reviews, see [36], [37]). In male *C. atrox* (adult min SVL  =  600 mm; [38]), spermatogenesis is initiated in spring and completed by late summer or early fall, and this sperm cohort is stored in the ductus deferens and used in the first mating season (late summer and fall), as well as the second mating season in spring that immediately follows hibernation (see [22], [23]).

At the SMs, the first mating season commences in mid- to late August, with males searching for and attending females, followed by courtship and coitus in early September, persisting through October. At SMs, our earliest observation of copulation is 2 September, and the latest is 15 October. Adult females typically undergo skin shedding (ecdysis) prior to or during the first mating season, whereas males typically shed from late October through November, at or near their den sites (G. W. Schuett & R. A. Repp unpubl. data). Unlike many rattlesnakes, female *C. atrox* from the SMs and nearby areas do not undergo major vitellogenesis in late summer and autumn; rather. females enter hibernation in November with small follicles [22], [23], [37], [39]. The period of sexual inactivity (hibernation) lasts about 120 to 130 days, from late October to early March.

The second mating season commences in mid-March, and persists to mid-May. In individuals that den (hibernate) communally, courtship, copulation, and male-male fights occur at or near the dens from mid- March to early April [22], [23] (G.W. Schuett & R.A. Repp, unpubl. data). After egress and mating in early spring, ova undergo rapid development (vitellogenesis) and ovulation occurs in late spring (May). Following the second mating season, adults of both sexes undergo ecdysis, typically from late May through June (G.W. Schuett & R.A. Repp unpubl. data). Births occur from early August to mid-September [22], [23], [39], but are centered in mid- to late August [23].

### Sexual behaviour, male fighting, and coitus

**Figure 1:**
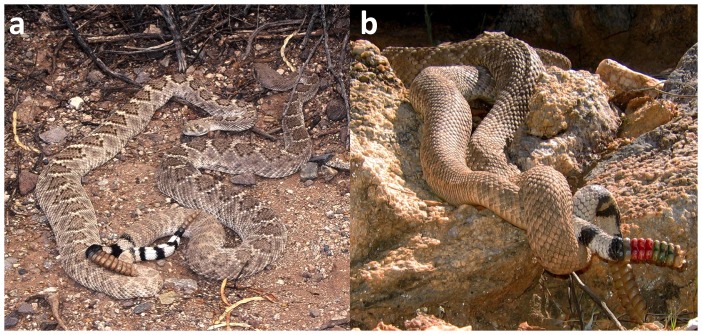
Sexual behavior in *Crotalus atrox*. (a) Pair of *C. atrox* in coitus. Unknown male (left) with female CA-3, September 13, 2001. (b) Pair of *C. atrox* in courtship below a den site. The lower body and tail of unknown male is wrapped over and around tail of female CA-44 (painted rattles), March 2012. Photographs by Roger Repp.

Like all snakes, male *C. atrox* are the mate-seeking sex [40]-[42], and courtship by males involves stereotypic behaviours [43] (G.W. Schuett & R.A. Repp unpubl data). In rattlesnakes, including *C. atrox*, attendance and courtship in nature can be protracted [41], [44], requiring days or even weeks before mating is effected. Coitus in *C. atrox* lasts 24 h or longer [28], [43] (GW Schuett & RA Repp, unpubl. Data; Figure 1). Wild males do not mate multiply with the same female in a single breeding season (G.W. Schuett & R.A. Repp unpubl. data). Male *C. atrox* engage in ritualistic, physical combat (without use of venom) for priority-of-access to females during the mating seasons [45], [46], and larger males tend to win fights and maintain dominance [43], [47] (G.W. Schuett & R.A. Repp unpubl data).

### Frequency of female reproduction and operational sex ratio (OSR)

Many female viperid snakes are long-lived (> 20 years), iteroparous, and show patterns of reproduction that are less than annual [39]-[41]. But recent work on *C. atrox* at the SMs has demonstrated that while a biennial pattern is typical for females, annual reproduction is not uncommon [22], [23]. The adult sex ratio at SMs appears to be at unity (G.W. Schuett & R.A. Repp unpubl data) and the operational sex ratio (OSR), which is the ratio of sexually competing males to sexually active females, is roughly 2:1 owing to the female reproductive patterns (i.e., skipping reproduction). The OSR is an important metric in sexual selection analyses [40], [41], [48]-[50]. When the OSR is male-biased, and hence fewer opportunities for males to obtain mates, sexual selection is predicted to act more strongly on males [40], [41].

### Determining female reproductive status

In each year subjects were radio-tracked and assigned a reproductive status of either pregnant (when they produced a litter) or non-pregnant. Following parturition, the reproductive status of a female changed to postpartum through the end of that year, and in the following year was re-assigned as either pregnant or non-pregnant. Like their viper relatives, female *C. atrox* are noticeably robust when pregnant [22], [23], [34], [39]. Thus, based on their increased mass, we were able to readily detect reproductive from non-reproductive females by mid- or late June. Births occur from early August to mid- September at sheltered sites, such as packrat (*Neotoma albigularis*) middens (nests), small mammal burrows, or rock shelves, but generally not at or near winter refugia [22], [23], [51]. Parturition was deemed imminent when female movements from these sites were greatly reduced or ceased; thus, radio-tracking efforts were typically increased to 1-2 times per day to better pinpoint birth dates. The number of offspring observed (or their molts) determined litter size. All mothers remained at birth sites until their progeny underwent their natal ecdysis, which occurred 5 to 7 days post-birth [22], [23]. Maternal attendance has been described in other populations of *C. atrox* [52], and it is a common feature in most species of rattlesnakes and other pitvipers of temperate North America [53], [54].

### Capture and immediate processing

Subjects were captured using conventional snake hooks and plastic grabbers. The capture process, which involved grabbing a snake and placing it into a clear plastic tube for temporary restraint (1 m length; diameter varied in size), was done gently and typically required less than 1 min to minimize handling stress [55]. In many cases, individuals were located basking at or near the entrances of dens in spring (March-April). At the time of capture Global Positioning System (GPS) coordinates were collected as Universal Transverse Mercators (UTMs).

Within 24-h of the initial capture, subjects were measured (snout-vent length, tail length, head dimensions to the nearest 1-mm; body mass to the nearest 1.0 g) and sex confirmed (via probing) while under anesthesia (isoflurane). For identification purposes, individuals were photographed, implanted with a unique PIT-tag (AVID, Inc., Norco, California, U.S.A.), and their proximal rattle segments were colored using Sharpie pens. In subjects selected for radio-tracking, each had an appropriately sized (< 5% of the total body mass; [56]) temperature-sensitive radio-transmitter (models SI-2T and AI-2T, 11-16 g; Holohil Inc., Carp, Ontario, Canada) surgically implanted within the coelom following general procedures used for snakes [22], [23]. After processing, all subjects were released at the exact site of capture.

### Tissue Sampling

All adult subjects had small amounts (0.1-0.3 ml) of blood harvested from caudal vessels for DNA parentage and kin analyses [23]. All blood was preserved in 95-100% ethanol, stored cold (0-10 C) and in darkness. Similar to other rattlesnakes [53], [54], neonates (littermates) of *C. atrox* remain with their mother until they shed their natal skin (4-7 days) and disperse [22] (G. W. Schuett & R. A. Repp unpubl. data). Because neonates are often difficult to sample without causing extensive damage to birth site microhabitat, we primarily used noninvasive sampling procedures to collect DNA from them. This was achieved by returning to birth sites (using GPS coordinates) after their post-shed dispersal and collected sheds found at or near the entrance of the birth site. Also, we opportunistically collected all other shed skins (all adults) found in the field, determining size and estimating sex of individuals by measuring the shed skin (SVL and tail length) and counting the subcaudal scales.

### Genotyping

We extracted DNA from blood and scale tissues using a salt extraction protocol and from shed skins using Qiagen DNEasy Tissue Kits (QIAGEN). We genotyped all individuals using 27 of the 30 microsatellite markers that amplified in *C. atrox* in Pozarowski et al. [57] and used their same genotyping methodology.

We excluded three of the loci that showed significant levels of linkage disequilibrium in their analysis (Ca2_23, Crti47, and Scu07) [57]. These markers included 13 (Ca1_14, Ca1_20, Ca1_22, Ca1_31, Ca1_39, Ca1_43, Ca2_27, Ca2_38, Ca2_64, Ca2_71, Ca2_74, Ca2_81, Ca2_90) developed by Pozarowski *et al.* [57], two (Crti09, Crti10) developed by Goldberg *et al*. [58], six (Crti12, Crti14, Crti23, Crti32A, Crti37, Crti95) developed by Munguia-Vega *et al.* [59], three (CwA14, CwA29, CwB6) developed by Holycross *et al.* [60], two (MFR15, MFR23) developed by Oyler-McCance *et al*. [61], and one (Scu05) developed by Gibbs *et al.* [62].

PCR amplification and genotyping procedures follow Pozarowski *et al.* [57]. All forward primers were 5′ end labeled with a tag (5′-GGAAACAGCTATGACCATG-3′) for tailed PCR with an M13 primer labeled with a 6-FAM, HEX, NED or PET (Invitrogen and Applied Biosystems fluorophores) [63]. All reverse primers were 5′ end labeled with a PIG-tail (5′- GTTTCTT -3′) to prevent adenylation [55], [64].

Microsatellite loci were amplified in 10 µl reaction volumes containing: 1.0-3.5 µM MgCl, 0.8 µM dNTPs, 0.2 µM forward M13 primer, 0.4 µM reverse primer, 0.4 µM M13 fluorescently-label, and 0.1units Platinum Taq DNA polymerase (Invitrogen) and 10ng of template DNA. We used an initial 3 min denaturation at 94°C, followed by 35 cycles of denaturation (94°C for 30s), annealing (50°C-62°C for 30s), and extension (72°C for 30s) with a final extension of 3 min at 72°C. Amplified microsatellite loci were pooled according to size and fluorophores followed by fragment analysis with an ABI 3130xl sequencer. To score microsatellite alleles we used the genotype analysis software GeneMarker v1.85 (SoftGenetics, State College, PA, USA).

### Spatial analyses

To perform spatial analyses, all UTM coordinates were transferred into ArcView 3.2 Spatial Analysis software (Environmental Systems Research Institute, Inc) and movement parameters were analyzed using the Animal Movement extension. Portions of these spatial data for females have been presented elsewhere [23,23]. We performed three different types of spatial analyses to examine relationships between male proximity and parentage: home range size, pairwise home range overlap between all individuals that were radio-tracked for at least one full year, and geographic midpoint comparisons.

Although kernel density estimators are commonly used for determining home range sizes of endotherms, recent analyses indicate they may not perform as well as minimum convex polygons (MCPs) for snakes and other herpetofauna [22], [23], [65]. Thus, we used ArcGIS 3.2 to compute 95% MCPs for each adult individual radio-tracked. These MCPs represent the smallest polygon that incorporates 95% of the relocations for an individual. To produce a single value for the degree of overlap for each pair, we calculated the average overlap for the two individuals in each pair as (AB/A + AB/B)/2, where A is the home range size of individual A, B is the home range size of individual B, and AB is the area shared by both A and B. Using this method we generated a pairwise matrix of average home range overlap values that could be compared to the probability of a male and a female sharing parentage in a litter.

Although we only had enough relocation data to calculate home ranges for the subset of individuals that had radio-transmitters, we computed the geographic center of all capture and recapture locations for all individuals using the online calculator Geo Midpoint (www.geomidpoint.com). This gave us a single location that represented the geographic center of all spatial locations where that individual had been recaptured over the course of our decade of fieldwork. We used the Geographic Distance Matrix Generator (biodiversityinformatics.amnh.org) to compute pairwise distances between geographic midpoints of all adult snakes. Because individuals use distinct spatial locations during summer (active home range) and winter (inactive overwintering range), we computed separate geographic midpoints for summer (May – September) and winter (October – April) relocations [23], [51].

### Parentage analyses

We used the software COLONY 2 [66] to examine the relationships among litters of neonates and identify any potential mothers or fathers of those litters within our field sample. COLONY 2 is well suited for this analysis because our data set contains several clusters of individuals known to be siblings (e.g., neonates from the same litter), often with a mother identified from field data. COLONY 2 uses a full-pedigree likelihood approach to estimating parentage, jointly considering both sibship and parentage relationships. By examining data from multiple offspring simultaneously, the probability that both parental alleles are represented increases, leading to more accurate parentage inference. COLONY 2 also has robust genotyping error models that can account for a relatively high frequency of genotyping errors both from allelic drop out and other sources [67]. Furthermore, a recent analysis using simulated data found that COLONY 2 outperformed other popular parentage inference methods, and was highly accurate with the use of 15 or more polymorphic markers [68].

In order to maximize the ability of COLONY 2 to assign parentage in our data set, we first determined locus-specific error rates for the remaining 27 loci. We used the program MICROERRORANALYZER, which implements the likelihood error estimates detailed in Wang [69], to estimate rates of null alleles, allelic dropout, and false alleles in a data set containing known parent-offspring dyads. We discarded loci with combined estimated error rates >20%, leaving us with a total of 18 loci. We then used the locus-specific error rates for these 18 loci in COLONY 2. To assess the ability of COLONY 2 to assign parentage in our sample, we first analyzed our data without assigning sibships to their known mother. Because COLONY 2 was able to identify the field mother as the genetic mother in all cases, we assumed that the likelihood estimation approach used in the program was robust with respect to our data set. In subsequent analyses with COLONY 2 we assigned sibships to known mothers to increase the information available for paternity assignments, and then retained only those paternity assignments with a maximum likelihood probability > 0.95. We conducted two separate runs with our data, and because the parentage assignments were identical across runs, we used these parentage assignments in all subsequent analyses.

### Male reproductive success and body size

We compared the body size (snout-vent length, SVL) of males siring progeny (paternity) to those with no detected paternity. Because not all males were measured in the year of which they were deemed fathers or were attending-courting females, we used a corrected estimate of SVL for the year in which parentage was documented (see [11]). Our corrected estimate was based on growth rate estimated from capture-recapture data of adult males at our site. We found males between 700-900 mm SVL increased in length (average) 35 mm per year, but male growth slowed at about 900 mm SVL, and males greater than 900 SVL increased in length (average) 11 mm per year. Thus, our corrected estimate of SVL used the closest year in which we measured the SVL of a male, and then adjusted that SVL up or down in a size-specific manner to reflect the number of intervening years between the paternity event and the measurement year.

### Male reproductive success and spatial analyses

We examined the effects of home range size and spatial proximity to mothers (home range overlap and midpoint distances) as factors affecting paternity. For home range size, we compared the average home range size of all males that were identified as fathers in any litter to all males that were not identified as fathers. For spatial proximity, we compared pairwise measures of spatial proximity between the males and female pairs who had parentage in litters to male and female pairs who did not share parentage. The distributions of pairwise values within groups that contain many individuals that do not share parentage do not conform to a normal distribution (the median and modal values of such distributions are usually 0), which makes traditional parametric statistics inappropriate. Thus, we used bootstrap resampling procedures to compare average home range overlap for male-female pairs that share parentage to randomly generated male-female pairs. For each comparison, we generated 1000 samples of random pairs via bootstrapping, with each sample equal in size to the focal group sample size. We calculated two-tailed p-values from the largest confidence interval around the mean of the resampling distribution that did not contain the mean of the focal group. We used the program RESAMPLING STATS for these procedures [70].

We used our field records of male-female pairings to determine the number of times different males were observed in the field paired with females during the mating season, as well as the proportion of times females who produced litters that paired with fathers or non-fathers. Because *C. atrox* exhibits two mating seasons (late summer-early fall and spring) prior to ovulation, we counted pre-birth pairings that occurred in both the fall and spring mating seasons preceding births. Also, we used our field records to compare the sizes (corrected SVL, see above) of males that were found attending females.

### Data Analysis

Unless noted otherwise, all statistical analyses were conducted with SYSTAT 12. Mean values are given as mean ± standard deviation. Before performing parametric tests, data were tested for the assumptions of normality and equal variances [71]. If data violated assumptions of normality or equal variances after transformations, we used non-parametric tests.

Genotyping data, morphological data, and spatial data (viewable via Google Earth) for all individuals are available from the public website, The Copperhead Institute (http://www.copperheadinstitute.org).

## Results

### Tissue sampling

We collected tissue samples from a total of 324 individuals. Of these, 25 did not produce DNA of sufficient quality for genotyping, leaving us with a total sample of 299 individual genotypes. Of these samples, 191 came from adult individuals sampled from 2001-2010. The other 108 samples came from neonates from 30 different litters produced by 18 different females [32]. Twenty-four of these litters were, to the best of our knowledge, complete litters, whereas only one individual was sampled from the remaining six litters. Mean litter size was 4.3±1.9 neonates. Although the mean litter size in our population is small relative to other regions, both litter size and adult body size vary geographically for this species [32], [34], [39]. Our findings of small and sometimes frequent (e.g., annual) litters are consistent with past research done at this and nearby sites [22], [23], [39], [72].

### Radio-tracking

We surgically implanted radio-transmitters in 26 adult females and 20 adult males, which we radio-tracked for a minimum of 1 year; many subjects were radio-tracked for several consecutive years, with a maximum of 7 consecutive years (Table 1). We collected detailed data on space use, mating behavior, and conspecific associations for these 46 individuals, as well as opportunistic data from stochastic sampling and re-sampling of 145 additional adult individuals.

**Table 1:** Summary of individuals tracked via radio-telemetry.

ID	Sex	Years tracked^1^	Home Range Size (hectares)
CA-1	F	7	9.5
CA-47	F	7	14.1
CA-2	F	6	5.3
CA-30	F	6	4.0
CA-16	F	5	3.5
CA-46	F	5	3.8
CA-61	F	5	32.7
CA-14	F	4	11.5
CA-44	F	4	4.6
CA-58	F	4	1.8
CA-102	F	3	8.9
CA-29	F	3	8.1
CA-42	F	3	2.7
CA-49	F	3	1.6
CA-64	F	3	1.1
CA-81	F	3	8.4
CA-93	F	3	2.5
CA-95	F	3	1.3
CA-100	F	2	5.7
CA-39	F	2	9.7
CA-59	F	2	5.0
CA-66	F	2	1.9
CA-94	F	2	0.7
CA-124	F	1	3.2
CA-62	F	1	0.3
CA-77	F	1	4.4
CA-6	M	4	17.2
CA-13	M	3	24.8
CA-31	M	3	32.1
CA-32	M	3	24.4
CA-33	M	3	14.9
CA-5	M	3	12.3
CA-117	M	2	8.5
CA-34	M	2	8.7
CA-50	M	2	42.3
CA-7	M	2	4.4
CA-76	M	2	5.1
CA-79	M	2	10.7
CA-96	M	2	23.1
CA-97	M	2	18.2
CA-98	M	2	22.0
CA-37	M	1	9.5
CA-38	M	1	1.6
CA-4	M	1	0.9
CA-55	M	1	37.9
CA-92	M	1	14.0

1 Indicates the total number of consecutive calendar years in which an individual was followed via radio-telemetry.

### Genetic parentage

We sampled 108 neonates, of which 105 (97.2%) were associated with a marked female (“field mother”) who was presumed to be the parent (Table 2). In all cases, COLONY 2 correctly identified the field mother as the genetic mother of those offspring. Additionally, COLONY 2 was able to assign the 3 “orphan” neonates in our study with no field mother as offspring of sampled females. COLONY 2 assigned the 108 sampled neonates to 27 different fathers. Of these fathers, 18 (66.7%) were known (pit-tagged) subjects. The other 9 fathers were not marked but could be identified as individual genotypes by COLONY 2. We detected multiple paternity in exactly half of the complete litters we sampled (12 of 24 litters, 50%). Five of the 12 litters had three fathers, and two of these cases involved all three offspring in the litter being sired by different fathers (Table 2).

**Table 2:** Summary of genetic parents identified for all litters of genotyped neonates.

Litter size^1^	Mother	Number of fathers	Identity of fathers^2^^,^ 3	Number of pairings^4^	Paired males with paternity^5^
1	CA-113	--	CA-50	na	na
1	CA-44	--	CA-74	1	1
1	CA-61	--	CA-27	2	0
1	CA-66	--	CA-50	na	na
1	CA-1	--	CA-50	2	0
1	CA-102	--	CA-68	0	0
2	CA-44	1	CA-68	na	na
2	CA-47	1	UM 2	na	na
2	CA-94	1	CA-97	1	1
3	CA-1	1	CA-43	0	0
3	CA-42	1	UM 8	1	0
3	CA-1	1	CA-27	3	0
3	CA-46	1	CA-68	2	1
3	CA-47	1	UM 5	1	0
4	CA-30	1	CA-45	1	0
5	CA-58	1	UM 2	0	0
7	CA-30	1	CA-84	0	0
9	CA-63	1	UM 1	na	na
3	CA-46	2	CA-9, UM-10 (2)	na	na
3	CA-58	2	CA-76, CA-20 (2)	2	0
4	CA-102	2	UM 9 (3), UM 10	0	0
4	CA-93	2	CA-73 (2), CA-5 (2)	1	0
5	CA-2	2	CA-23 (2), CA-5 (3)	3	2
6	CA-124	2	CA-108 (5), CA-74	0	0
9	CA-113	2	CA-80 (5), UM 13 (4)	na	na
3	CA-42	3	CA-76, UM 9, UM 10	0	0
3	CA-81	3	CA-45, CA-88, UM-8	1	1 possible
5	CA-1	3	UM 9, UM 14, UM 15 (3)	1	0
5	CA-47	3	CA-40, CA-45 (2), CA-5 (2)	0	0
6	CA-16	3	CA-43 (2), UM 2 (2), UM 10 (2)	2	0

1 Litter sizes of one indicate a sole neonate found after dispersal from birth site.

2 CA  =  *Crotalus atrox,* UM  =  Unmarked male.

3 Parentheses after male names indicate total number of offspring fathered in litter when that number is greater than one.

4 Number of males that female was found paired with during the fall and or spring mating period preceding parturition. NA (not applicable) indicates female was not telemetered prior to parturition.

5 Of those males found paired with females in the preceding fall and or spring mating periods, the number that were genetic fathers of any offspring in the litter. See text for details.

### Male body size and reproductive success

**Figure 2:**
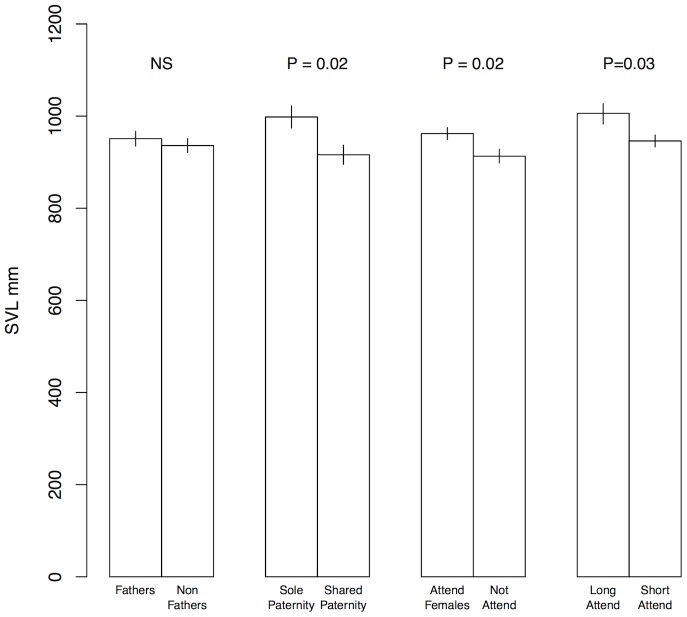
Size comparisons among males. Comparisons show average size and standard error of males identified as genetic fathers versus those that were not (fathers versus non-fathers), males that were the only father identified in a litter versus those that shared paternity with other males (sole paternity verus shared paternity), males that were seen attending females during the mating season versus those that were not seen to do so (attend females versus not attend), and males that attended females for longer than one week versus those attending females less than a week (long attend versus short attend).

Males we identified as fathers did not differ significantly in SVL from males with no detected paternity (mean SVL fathers: 951±86 mm; mean SVL non-fathers: 936±104 mm, *T* = 0.7, *p* = 0.48) (Figure 2). Of the 24 litters we sampled that had more than one neonate, a single male sired 12 of them and the remaining 12 had two or three fathers (Table 2). Marked males that did not share paternity (single sires of litters) were significantly larger than marked males that shared paternity (mean SVL single paternity: 998±63 mm; mean SVL shared paternity: 916±89 mm, *T* = 2.6, *p* = 0.02) (Figure 2). However, there was no correlation between total number of offspring fathered and body size (Pearson Correlation, *r* = - 0.27 *p* = 0.14).

### Male attendance, mating, and parentage

Males were often found attending females during the two mating seasons, occasionally for long periods (e.g., weeks). Attendance of females was influenced by male body size (Figure 1). Males that were found in attendance with females were larger in SVL than males that were not observed attending females (SVL attending males: 962±87 mm; SVL non-attending males: 913±107 mm, *T* = 2.4, *p* = 0.02) (Figure 2). Males that attended the same female for more than one week were significantly larger than males found in attendance for less than one week (SVL prolonged attendance: 1005±71 mm; SVL single attendance: 946±73 mm, *T* = 2.2, *p* = 0.03) (Figure 2).

Male attendance and mating behavior, however, was not strongly associated with male parentage. Of the 30 litters in our sample, 23 (76.7%) were from females that were being tracked by radio-telemetry prior to parturition. In 15 of these 23 litters (65.2%), females were found paired with one or more males in one or both mating seasons that preceded parturition. Of the 24 male-female pairings, females were paired with one male in 8 cases, two separate males in four cases, and three separate males in two cases. Of these 24 male-female pairings, 18 of them (75%) were with males that did not have paternity in the litter, and only 5 of them (21%) were with males that were positively identified as fathers (the remaining pairing was with an unmarked male with uncertain paternity, Table 2). Three of the pairings persisted beyond 7 days, and of these three cases, one male was the sole father of the subsequent litter and the other two males had no paternity in the subsequent litters.

### Spatial analyses

Our sample of adult male snakes that were radio-tracked for at least one active season (N = 20) included four individuals identified as fathers. These four fathers did not have significantly larger home ranges than the 16 radio-tagged non-fathers (identified fathers: 19.5±16.1 hectares; non-fathers: 15.7±10.9 hectares, *T* = 0.64, *p* = 0.68).

We found a total of seven unique male-female pairs that shared parentage in a litter and were both radio-tracked for at least one active season. These seven pairs had an average proportion of home range overlap of 0.30±0.26, which was significantly greater than the home range overlap of random male-female pairs from the population (resampling distribution 95% CI = 0 – 0.23, *p* = 0.008).

In addition to home range overlap, we compared the distance between the geographic midpoints of the capture locations of male-female pairs that shared parentage. We compared active season midpoints separately from overwintering midpoints. For the active season, we found a total of 28 unique male-female pairs that shared parentage in a litter. These pairs were, on average, 439±331 meters apart, which is significantly closer than the distance between random male-female pairs (resampling distribution 95% CI  =  446 – 661 m, *p* = 0.03). For the overwintering comparison, we found 24 male-female pairs that shared parentage and had overwintering midpoints. Overwintering sites were, on average, 287±248 meters apart for these pairs, which was also significantly closer than random male-female pairs (resampling distribution 95% CI  =  304 – 549 m, p = 0.016). This tendency of fathers to be in closer spatial proximity to females with which they shared parentage was not an artifact of these fathers being more centrally located at the study site. The average distance between male midpoints and the midpoint of all female midpoints was not significantly different for either active season relocations (fathers 385±195 m, non-fathers 405±157 m, *T* = 0.36, *p* = 0.72) or winter relocations (fathers 226±273 m, non-fathers 195±132 m, *T* = 1.19, *p* = 0.24).

### Female fecundity

The incidence of multiple paternity was not related to female fecundity, as the mean litter size for litters with single fathers (3.8±2.2) was not significantly different from the mean size of multiply sired litters (4.7±1.7, *T* = 0.9, *p* = 0.3). Females with multiply sired litters did not have larger home ranges than females with single-sired litters (home range size single sires: 6.3±4.7 ha; multiple sires: 5.8±3.9 ha, *T* = 0.1, *p* = 0.90).

## Discussion

### Sampling, genotyping and parentage analyses

We genotyped all adult and neonate snakes sampled in this study using a large number of polymorphic microsatellite loci markers and reconstructed parentage of the progeny by way of a full-pedigree likelihood approach. Most neonates were found in litters attended by solitary, post-partum females who were unambiguously assigned to be their mothers based on intensive radio-tracking [22], [23]. In female North American viperids, communal birthing is common [73], [74], but it is absent in *C. atrox* from our study site [22], [23]. Even when we exclude maternal information in the initial parentage analysis, COLONY 2 identified the attending female as the genetic mother of the sampled litter, indicating that the full-pedigree likelihood method is robust with respect to determining parentage in our samples [66]-[69], [75]. This step was important to confirm that adult females found in attendance with newborn litters were the true genetic mothers of those offspring, given recent genetic evidence that communally gestating midget-faded rattlesnakes (*Crotalus concolor*) have been found in close association with neonates that were not their progeny in so-called nursery aggregations [76].

Although blood was obtained in several instances from neonates, most DNA was derived from shed skins left by dispersing neonates 4-7 days after birth [31], [32]; thus, owing to various levels of probable environmental degradation of these delicate sheds, many of our markers had high levels of allelic dropout. These genotyping errors would almost certainly preclude exclusion methods for paternity assignment. In many studies using noninvasive tissue sampling, error rates are calculated by comparing replicate genotypes obtained from multiple PCRs using the same DNA sample (i.e., the “multi-tube” approach [77]. However, none of the replicate genotypes are guaranteed to be error-free, and so the consensus of a limited number of replicates could still contain errors [67]. For likelihood-based parentage analysis, it is still necessary to estimate an error rate of the consensus genotype. The error rate estimated from comparing replicate runs is the frequency of inconsistent genotypes per replicate, not the error rate of the consensus genotype [69]. Thus, even if replicate genotypes were constructed, some non-PCR based method of estimating genotyping error would still be desirable. If samples contain known parent-offspring dyads, genotyping error rates can be estimated from pedigree analysis using maximum likelihood approaches. We chose to use this approach, given the relatively large number of known mother-offspring pairs in our sample. Using a locus-specific maximum likelihood error estimation approach allowed us to effectively use the full-pedigree likelihood approach to estimate parentage with confidence. This approach has utility for other vertebrate taxa, since noninvasive tissue samples can often be reliably collected from groups of known sibships (i.e., tissues from shed skins, hatched eggshells, or litters attended by mothers, [78]).

Our analysis identified 18 males in our sample as fathers of 64 neonates; unmarked ( =  unknown) males sired the remaining 44 neonates. Twenty-three of the litters we genotyped were from females being actively radio-tracked in the year prior to parturition. Even though we were able to observe these females courting or mating on several occasions, only 25% of the males that paired with these females proved to father offspring in subsequent litters. Despite the fact that we were unable to assign a known (marked) father to a large proportion of neonates, the use of highly informative microsatellite markers was invaluable for estimating reproductive success. In snakes, owing to a range of possible mechanisms (e.g., polyandry, long-term sperm storage, cryptic female choice, sperm competition), strict behavioral observations are poor indicators of paternity [9], [20], [79]. In the present study, although molecular results bore out our behavioral observations of female reproduction (i.e., mother-progeny associations), estimates of paternal contributions would have been highly inaccurate had we relied solely on behavioral observations. Thus, recent triumphs in the study of reproductive success and mating systems in secretive species, such as snakes and other vertebrates, have been made possible over the past several decades by the revolutionary advancements in parentage analysis via microsatellite genotyping [6], [9], [11], [12], [14], [21].

### Multiple paternity

Our behavioral observations and molecular results indicate that both male and female *C. atrox* copulate with multiple partners in one or both of the mating seasons, and that multiple paternity in litters consist of 2 to 3 fathers (reviewed in [79]). However, successful long-term sperm storage (LTSS) by females, which is documented in *C. atrox*, confounds our ability to precisely pinpoint the temporal estimates of copulations [35], [80]-[82]. In other words, although we are confident of the methods used to assign fathers to particular litters, copulations might have occurred outside of the years we sampled, perhaps even by snakes that had mated and died [83]. Schuett *et al.* [55] demonstrated successful LTSS in a field-collected *C. atrox* in autumn 1999, and held in laboratory isolation from all other snakes. That subject produced a healthy litter of 3 males and 3 females on 28 August 2000. In summer 2002, this female produced another healthy litter (4 males, 5 females) despite the fact that she remained in strict isolation (G. W. Schuett unpubl. data). In the second litter, spontaneous facultative parthenogenesis was ruled out owing to certain litter characteristics (see [82]).

Although the mean litter size was relatively small in the present study with 4.3 neonates per litter (see [39]), half that were sampled were multiply sired, with 21% having three fathers. This fits the broad pattern of high levels of multiple paternity found in other snakes [79] and other squamate reptiles [84], including two other viperid species where researchers have tested for multiple paternity of wild litters using molecular techniques [11], [84], [85].

Paternity was unequally distributed across our sample of adult males. Most males we sampled (77%) were not detected as fathers in our samples, whereas a few individuals (e.g., CA-50) had paternity in several litters (Table 2). Highly skewed distributions of paternity are typical in species with polygynandrous mating systems, which is believed to lead to sexual size dimorphism (SSD) [86]. However, we found no difference in the mean size of fathers and non-fathers in our sample, despite that fact that this species exhibits male biased SSD and male-male combat. This result may be due in part to the fact that our methods do not allow us to identify with certainty males that did not mate; certainly, non-fathers could have sired progeny in litters that were not in our samples.

### Male body size and reproductive success

Male-biased SSD is documented in *C. atrox* [28], [30], [87], [88], though it is variable in degree among populations in Arizona [32]. Nonetheless, whether SSD in *C. atrox* is strictly an environmental outcome, influenced by sexual selection, or both, remains to be tested in future investigations. Although our analysis was unable to show that fathers were larger than non-fathers, which may be due to sample size (i.e., individuals could have paternity in unsampled litters), we found limited evidence for size-specific male mating strategies. Males that were the sole fathers of litters were larger (SVL) than males that shared paternity with other males. Field observations also indicated that males found attending females in the mating season were larger than males that we did not observe attending females. Furthermore, males that were found to attend females for prolonged periods (longer than one week) were larger still than males found attending females once or briefly. These patterns indicate that larger males are more likely to actively guard females, thus restricting access by other males. Although pre-copulatory mate-guarding behavior appears to lead to larger males being less likely to share paternity in a given litter, it should be noted that we found no overall relationship between the number of offspring fathered and male body size (reviewed in [79]).

Our results on male body size (SVL) are similar to previous studies that have investigated paternity in snakes with male-male combat. Blouin-Demers *et al.* [12] found that larger male black rat snakes sired more offspring than smaller males, and also sired a higher proportion of offspring per clutch. Ursenbacher *et al.* [11] found that larger male adders sired more offspring, and that single father litters were sired by larger males than multi-father litters. In a study that did not use molecular parentage methods, Madsen *et al.* [89] found that larger male adders dominated smaller ones in male combat bouts and tended to mate-guard females, but smaller males mated with females in the absence of other males. Dubey et al. [14] found that larger males of slatey-gray snakes (*Stegonotus cucullatus,* Colubridae) sired a greater proportion of offspring within a clutch than smaller males. In contrast, Weatherhead *et al*. [13] found that body size was not clearly related to reproductive success in northern water snakes, a species which lacks male-male combat. Also, Duvall & Schuett [52] showed that body size in male prairie rattlesnake (*Crotalus viridis*) was not related to mating success.

In snakes with male-male combat, body size is an important determinant of reproductive success, as would be expected given that male size is the primary determinant of winning male combats [47]. However, in all of the above examples, smaller males were also able to father some offspring: male adders as small as 37 cm, black ratsnakes as small as 81 cm, slatey-gray snakes as small as 85 cm, and western diamond-backed rattlesnakes as small as 70 cm SVL were all fathers. For adders, black ratsnakes, and western diamondback rattlesnakes, these sizes are on the lower end of the estimated minimum male size at sexual maturity [88], [90], [91]. Thus, even snakes with male-biased SSD and male-male combat may also exhibit alternative male mating tactics, whereby small males attempt to “sneak” copulations with females without engaging in combat with other males. Although data on alternative male mating tactics in snakes are limited, studies of adders [89] and garter snakes [92] indicate that small males may successfully employ different mating tactics than large males. Our data on paternity in *C. atrox* indicate that alternative tactics by smaller males may be effective, as there was no overall relationship between body size and reproductive success.

### Spatial ecology of males and reproductive success

Our spatial analyses indicated that proximity of the male-female pairs was a significant factor associated with paternity in a given litter. This relationship was detected for both the active (March-October) and overwintering (November-February) seasons. On average, during the active season, fathers were captured within 450 meters of females that shared parentage with them. This distance is not unexpected, given that the average male home range in our population was 16 hectare (i.e., a 16 hectare circle would have a diameter of about 450 m). In support of this finding, we found a significant degree of home range overlap for those male-female pairs sharing parentage and were radio-tracked. Consequently, several different spatial analyses we performed indicated that males typically mate only with females that are likely to overlap some part of their home range [108], a finding that reinforces the general principle of spatial distribution of receptive females as a primary factor shaping mating systems [48]. Accordingly, we predicted that males with larger home ranges would also potentially mate with more females and sire more offspring. Although males who fathered offspring did not have significantly larger home ranges than males with no detected paternity, our sample size for this test was low, as only four genetic fathers had radio-transmitters. Given our data on the importance of spatial overlap for paternity, future analyses may reveal that males with larger home range sizes have greater relative reproductive success [109].

### Snake mating systems and the evolution of polyandry

Over two decades ago, the first modeling attempts identifying, characterizing and quantifying the mating systems of snakes within formal selection theory described most species as being polygynous based on the available empirical evidence [40], [41], [49], [80]. Since that time a wealth of new information on populations, behavior, reproduction, and parental care of snakes has emerged [53], [82], [93], [94]. Accordingly, adjustments need to be made to accommodate these discoveries and shifts in perspectives. Based on behavioral and genetic information we have for *C. atrox* at the SMs, we suggest that adults assemble as itinerant pairs during the breeding seasons, with a mating system characterized as attendant polygynandry (see [93], pp 274-280 in [95]). This breeding system has the following characteristics: (1) males seek females, which are unevenly distributed and not clumped (see [41], [96]); (2) the OSR is male-biased (roughly 2:1) in any given year; (3) bisexual pairs last for various lengths (e.g., days to weeks) but are not permanent; (4) male defense of females (male combat) may occur; and (5) both sexes can mate multiple times with multiple partners per breeding season.

Our empirical and theoretical understanding of multiple mating in females, sometimes termed polygamy, has undergone a revolutionary paradigm shift, beginning with Parker’s [97] highly influential paper on sperm competition in 1970. This work has spawned thousands of studies investigating male and female mating frequency, sperm competition, cryptic female choice, and multiple paternity in plants and animals [98], and has led to a great expansion of our knowledge and understanding of mating systems and sexual selection [49], [95], [99]-[101]. It appears straightforward why some males mate with as many females as possible, based on the vast majority studies and theoretical models examining fitness benefits [95], [102]. This has been termed the “Darwin-Bateman paradigm” [103], which asserts that the reproductive success of males increases steeply (Bateman gradient) as the number of copulations with different females increases [100]. Nonetheless, several important topics remain problematic, and perhaps the most persistent one concerns the adaptive significance of polyandry, defined as multiple matings with different males by females [95], [101]. Why should females mate with multiple partners for fertilization of a single clutch or litter, especially in cases where female fecundity does not seem to increase with multiple mating, as we found for *C. atrox* in this study?

Various hypotheses addressing the adaptive significance (direct and indirect benefits) of the evolution for and maintenance of polyandy have been proposed and tested. Recent interest has been explosive [104], [105], and this renaissance sets the stage for reexamining earlier research and executing new empirical and modeling studies that expand beyond sexual selection and sexual conflict. Adaptive conclusions regarding polyandry in animals are complex and inconclusive, with some studies revealing benefits and others reporting none [106], [107]. The study of polyandry, sperm competition, long-term sperm storage, and cryptic female choice is in its infancy with regard to studies of snakes [79], [84], [93]. For most species, it is difficult to assess whether multiple matings in wild females have indirect benefits, such as fertilization insurance, genetic compatibility, sperm competition, or increased genetic diversity (reviewed in [104]). Instead, long-term field studies will have to be combined with experimental manipulations to address these hypotheses empirically.

## Conclusions

Our use of genetic techniques to analyze parentage has allowed us to substantially increase our knowledge of the mating system of a population of western diamond-backed rattlesnakes (*C. atrox*) in the Sonoran Desert of Arizona. This species is similar to many other snake species in that they exhibit a “polygynandrous” mating system, with both sexes mating with multiple partners during the mating season or seasons. Larger males appear to use their larger body size to prevent other males from mating with females that they are guarding or have mated, but smaller males are able to successfully father offspring with females. Although molecular tools have now been used in a handful of snake species to assess parentage and multiple paternity [84], [85], [110], [111], their use in combination with long-term field studies remains quite limited. In *C. atrox* and other snakes, field observations of mating alone were insufficient to characterize the genetic mating systems, both because mating behavior is difficult to observe directly, and because the act of mating does not guarantee reproductive success in males (see [10]). Accordingly, we urge field researchers to adopt molecular analyses as a standard tool in long-term, individual-based studies of the ecology and behavior of snakes [1], and think that doing so will greatly improve and transform our understanding of mating systems, behavioral ecology and sexual selection of this large, diverse and important group of vertebrates.
